# Impact of restrictive versus liberal transfusion and clinical outcomes in critically ill children: A retrospective observational study

**DOI:** 10.1002/hsr2.898

**Published:** 2022-10-20

**Authors:** Saptadi Yuliarto, Kurniawan Taufiq Kadafi, Luluk Nur Azizah, William Prayogo Susanto, Takhta Khalasha

**Affiliations:** ^1^ Division of Pediatric Emergency and Intensive Care, Department of Pediatrics, Faculty of Medicine, Saiful Anwar General Hospital Universitas Brawijaya Malang Indonesia

**Keywords:** anemia, critical ill, length of stay, mechanical ventilation, mortality, RBC transfusion

## Abstract

**Background and Aims:**

Critically ill children with anemia often requires blood transfusion, which can cause several complications. It is important to decide when to start the red blood cell (RBC) transfusion; however, the guidelines is still lacking. The aim of this study was to compare restrictive and liberal transfusion strategy.

**Methods:**

This is an observational retrospective study of critically‐ill children who receive RBC transfusion. Subjects categorized into two groups by initial hemoglobin (Hb), that is, restrictive (Hb ≤ 7 g/dl) and liberal (Hb ≤ 9.5 g/dl) strategy. In each group, subjects categorized based on: (1) Hb increment: high (increased ≥2.5 g/dl) and low (increase <2.5 g/dl) and (2) final Hb level: low (<7.0 mg/dl), moderate (7.0–10.0 mg/dl), and high (>10.0 mg/dl). Patient with hematologic or congenital disorder, severe malnutrition, chronic infection‐related anemia, and transfusion in Hb level ≥9.5 g/dl were exclude. Each patients were evaluated for the clinical outcome, which is: intensive care length of stay (IC‐LOS), length of mechanical ventilation (LoMV), and mortality rate.

**Results:**

Clinical outcome and mortality rates of both transfusion strategies are similar. The mortality rates were lower in higher Hb increment and final Hb level (*p* = 0.04 and *p* = 0.01, respectively). Multivariate analysis in all groups revealed mortality rate had moderate correlation with Hb increment (odds ratio [OR] = 0.694, 95% confidence interval [CI] 0.549–0.878; *p* = 0.002) and moderate correlation (OR = 0.642, 95% CI 0.519–0.795; *p* = 0.000) with final Hb level. The similar results was found after categorization based on transfusion strategy.

**Conclusion:**

We conclude the restrictive and liberal transfusion strategy have a similar effect to IC‐LOS, LoMV, and mortality rate. High Hb increment (≥2.5 g/dl) and moderate‐high final Hb (≥7.0 g/dl) after transfusion reduce the mortality rate.

## 1 INTRODUCTION

Critical illness in children often results in anemia due to various mechanism, such as decrease of erythropoietin activity, excess usage of iron, or bleeding.^1^ Anemia could aggravate the patient's condition by reducing tissue oxygen delivery. Anemia incidence in children with critical illness who require pediatric intensive care unit (PICU) admission reaches 41% in 1–3 days, and around 74% of them required red blood cell (RBC) transfusion.^2^ Packed red cell (PRC) transfusion provides addition of hemoglobin (Hb) to increase blood oxygen capacity and restoration of intravascular volume to maintain cardiac output and organ perfusion.^3^

Massive transfusion might be demanded in certain situation of acute blood loss, but this treatment also brings some sort of complication. Acute blood loss causing acute anemia will induce platelet function disruption which ends on loss coagulopathy. Massive transfusion causing dilutional coagulopathy by activating extrinsic coagulation process and disrupt the hemostatic mechanism even further.^4^ The other harm caused is citrate overload that can significantly induce hypocalcemia and metabolic acidosis. This condition also contributes to alteration of hemostatic process.^5^ Another complication of transfusion also including transfusion‐related acute lung injury, allergic reaction and transfusion‐associated circulatory overload.^6^ Thus, the transfusion treatment must be decided carefully to achieve the purpose and minimize the complication.

Recently, none of the guideline fully describes the strategy of blood transfusion for children with critical illness. Restrictive transfusion could reduce the frequency of transfusion, which might be beneficial than liberal transfusion strategy. TRIPICU study compared restrictive transfusion (Hb ≤ 7 g/dl) and liberal transfusion (Hb ≤ 9.5 g/dl) in stable hemodynamic critically‐ill children showed no significant differences. Other studies by Cholette et al.^7^ compared these two transfusion strategies in children with cyanotic heart disease also reported no significant differences in circulation parameter. In pediatric sepsis population, early RBC transfusion may benefit to reduce multiple organ dysfunction days in more severe stage of septic shock.^8^ Furthermore, it remains questionable which the predictor for outcome is, the initial, increment, or final Hb level. The primary objective of this study was to compare restrictive and liberal transfusion strategy; whereas the secondary objectives were to compare Hb increment and final Hb level to clinical outcome of critically ill children.

## 2 METHODS

This is an observational retrospective study. Subjects was children aged 1 month–18 years old who had been hospitalized at high care unit (HCU) or PICU, and received PRC transfusion. Patient who had any hematologic or congenital disorder, severe malnutrition, chronic infection‐related anemia, and transfusion in Hb level ≥ 9.5 g/dl were excluded from this study. Subjects categorized into two groups based on initial Hb level, that is restrictive (Hb ≤ 7 g/dl) and liberal (Hb ≤ 9.5 g/dl) transfusion strategy. Then, in each group, subjects categorized based on: (1) Hb increment and (2) final Hb level. First, based on Hb increment, subjects divided into two groups, that is high (increased ≥2.5 g/dl) and low increment (increase <2.5 g/dl). Second, by the final Hb level, subject divided into three groups, low (<7.0 mg/dl), moderate (7.0–10.0 mg/dl), and high (>10.0 mg/dl). This study were conducted from January 1, 2015 to September 30, 2019 at HCU/PICU of Saiful Anwar Hospital. All the protocol in this study has already been approved by the Ethical Committee of Faculty of Medicine, Universitas Brawijaya, Indonesia (no. 400/256/K.3/302/2019).

Each patient was evaluated for the clinical outcome, that is, intensive care length of stay (IC‐LOS), length of mechanical ventilation (LoMV), and mortality rate. Descriptive data reported in median and interquartile range (IQR). To compare the outcome, each category was analyzed with univariate analysis by independent *t*‐test and *χ* ^2^ test (if normally distributed), or by Mann–Whitney and Fisher exact test (if not normally distributed). Variable with *p* < 0.25 included in multivariate analysis. The statistical analysis was performed by using SPSS software version 21 (SPSS Inc.) and the differences between groups considered statistically significant at *p* ≤ 0.05.

## 3 RESULTS

### 3.1 Subject characteristics

There were 352 critically ill children (1 month–18 years) with anemia who received PRC transfusion, during the period of 2015–2019. One hundred sixty‐seven subjects were excluded, because of hemato‐oncological disease (66 subjects), chronic disease‐related anemia or severe malnutrition (80 subjects), and transfusion in Hb level ≥ 9.5 g/dl (21 subjects).

**Figure 1:**
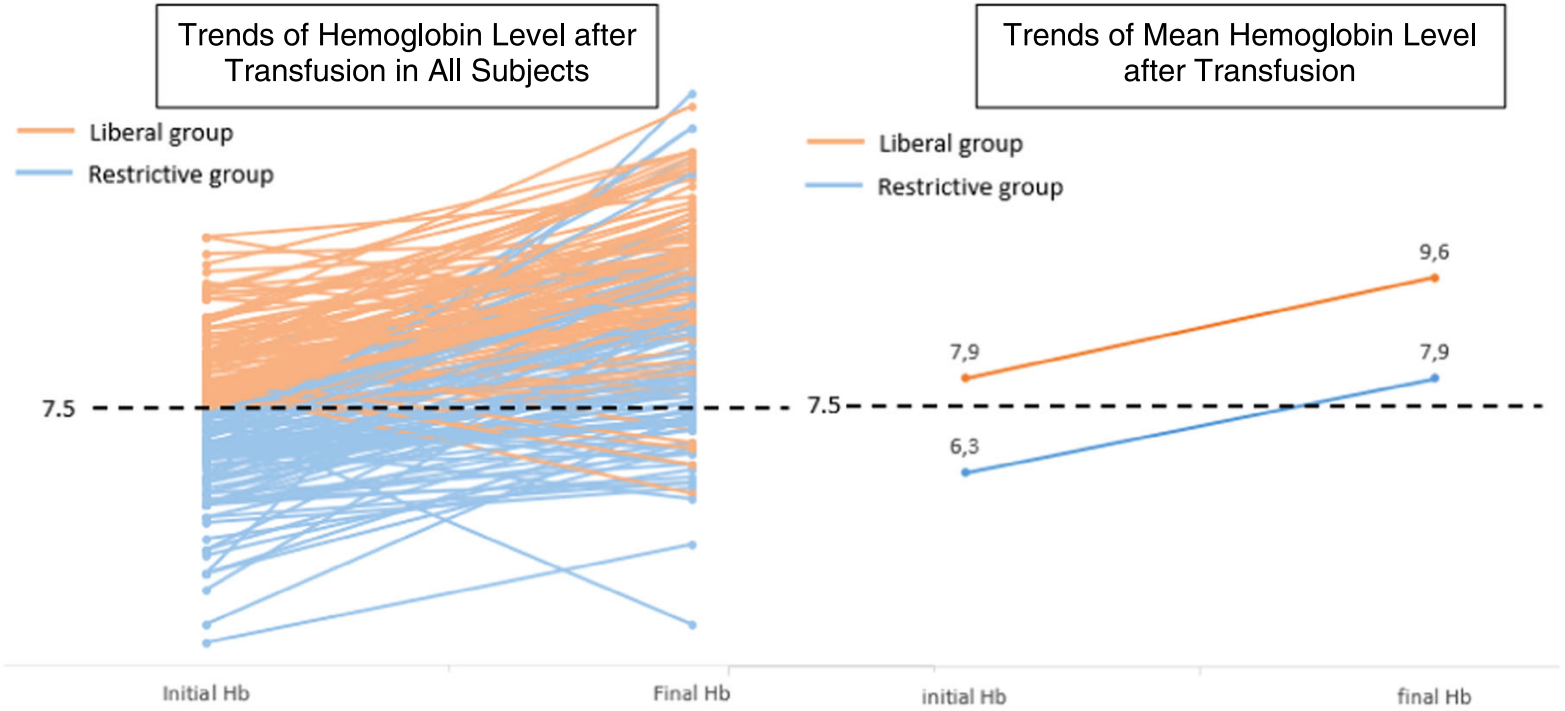
Trends of hemoglobin (Hb) level after transfusion in restrictive and liberal group. Left panel showed all subjects in restrictive (blue lines) and liberal (brown lines) group. Right panel showed mean Hb level in restrictive (blue lines) and liberal (brown lines) group.

From the total of 185 children included, 120 subject classified into liberal and 65 subject classified into restrictive transfusion group. Subjects in restrictive was older than liberal group. Male were more frequent in both groups. The median initial Hb level for transfusion threshold were 6.3 (IQR: 3.4–7) and 7.9 (IQR: 7.1–9.5) g/dl in restrictive and liberal group, respectively. Hb increment was 1.7 (IQR: −1 to +8.4) yielded final level 7.9 (IQR: 5.1–13) in restrictive group, whereas Hb increment was 1.5 (IQR: −3.6 to +4.5) yielded final level 9.6 (IQR: 3.7–12.8) in liberal group (Figure 1). PIM‐2 score was comparable between two groups (Table 1).

**Table 1:** Baseline characteristics.

Characteristics	Restrictive (*n* = 65)	Liberal (*n* = 120)
Age, months, median (IQR)	24 (1–180)	12 (1–204)
Gender, *n* (%)		
Male	41 (35.7)	74 (64.3)
Female	24 (34.3)	46 (65.7)
Hb level		
Initial, g/dl, median (IQR)	6.3 (3.4–7)	7.9 (7.1–9.5)
Increment, g/dl, median (IQR)	1.7 (−1 to +8.4)	1.5 (−3.6 to +4.5)
Hb increment categories, *n* (%)		
High (≥2.5 g/dl)	22 (33.8)	31 (25.8)
Low (<2.5 g/dl)	43 (66.2)	89 (74.2)
Final, g/dl, median (IQR)	7.9 (5.1–13)	9.6 (3.7–12.8)
Final Hb level categories, *n* (%)		
Low (<7.0 g/dl)	14 (21.5)	5 (4.2)
Moderate (7.0–10.0 g/dl)	46 (70.8)	69 (57.5)
High (>10.0 g/dl)	5 (7.7)	46 (38.3)
Diagnosis, *n* (%)		
Pneumonia	30 (16.2)	62 (34.6)
Postsurgical disease	30 (16.2)	42 (22.7)
Dengue shock syndrome	5 (2.7)	14 (7.6)
PIM‐2 score	0.82 (0.09–76.2)	0.75 (0.05–94.6)

Abbreviation: IQR, interquartile range.

### 3.2 Intensive care length of stay

The IC‐LOS was 4 (IQR: 1–18) days in restrictive group and 6 (IQR: 2–27) days in liberal group (*p* = 0.08). Based on Hb increment, the IC‐LOS was 5 (IQR: 1–24) days in low and 5 (IQR: 1–19) days in high increment group (*p* = 0.112). Based on final Hb level, the IC‐LOS was 3 (IQR: 1–9), 6 (IQR: 3–13), and 5 (IQR:3–11) days in low, moderate, and high group, respectively (*p* = 0.080) (Table 2).

**Table 2:** Clinical outcome based on all groups (before categorization based on transfusion strategy or initial Hb).

Categories	IC‐LOS, days, median (IQR)	*p* Value	LoMV, days, median (IQR)	*p* Value	Mortality rates, *n* (%)	*p* Value
Transfusion strategy (initial Hb level)						
Restrictive	4 (1–18)	0.08	1 (1–12)	0.18	40/65 (61.5)	0.07
Liberal	6 (2–27)		1 (1–15)		59/120 (49.2)	
Hb increment						
Low	5 (1–24)	0.11	1 (1–15)	0.62	78/135 (57.9)	0.04*
High	5 (1–19)		1 (1–16)		21/50 (42.0)	
Final Hb level						
Low	3 (1–9)	0.08	1 (1–7)	0.39	16/19 (84.2)	0.01*
Moderate	6 (3–13)		1 (1–9)		61/115 (53.0)	
High	5 (3–11)		2 (1–6)		22/51 (43.1)	

Abbreviations: Hb, hemoglobin; IC‐LOS, intensive care length of stay; IQR, interquartile range; LoMV, length of mechanical ventilation.

*Significant at *p* ≤ 0.05 level.

If Hb increment was categorized based on transfusion strategy or initial Hb level, in the restrictive strategy IC‐LOS was 5 (IQR: 2–8) days in low and 3.5 (IQR: 3–10.25) days in high increment group (*p* = 0.413); whereas in the liberal strategy, the IC‐LOS was 6 (IQR: 3–14.75) days in low and 6 (IQR: 3–12.5) days in high increment group (*p* = 0.590) (Table 3).

**Table 3:** Clinical outcome based on hemoglobin (Hb) increment and final hemoglobin level after categorization based on transfusion strategy or initial Hb.

Transfusion strategy	Categories	IC‐LOS, days, median (IQR)	*p* Value	LoMV, days, median (IQR)	*p* Value	Mortality rates, n (%)	*p* Value
Restrictive	Hb increment						
	Low	5 (2–8)	0.413	1 (1–3)	0.911	29/43 (67.4)	0.171
	High	3.5 (3–10.25)		1 (1–3)		11/22 (50)	
	Final Hb level						
	Low	2.5 (1–8.25)	0.11	1 (1–3.75)	0.844	11/14 (78.6)	0.078
	Moderate	5 (3–10)		1 (1–3)		28/46 (60.9)	
	High	3 (2.5–11)		1 (1–6.5)		1/5 (20)	
Liberal	Hb increment						
	Low	6 (3–14.75)	0.59	1 (1–5)	0.358	49/92 (53.3)	0.104
	High	6 (3–12.5)		2 (1–7)		10/28 (35.7)	
	Final Hb level						
	Low	6 (1.5–16)	0.382	1 (1–10)	0.567	5/5 (100)	0.072
	Moderate	7 (3.5–15)		1 (1–5.5)		33/69 (47.8)	
	High	5.5 (3.5–11.25)		2 (1–6.25)		21/46 (45.7)	

Abbreviations: IC‐LOS, intensive care length of stay; IQR, interquartile range; LoMV, length of mechanical ventilation.

If final Hb level was categorized based on transfusion strategy or initial Hb level, in restrictive strategy IC‐LOS was 2.5 (IQR: 1–8.25), 5 (IQR: 3–10), and 3 (IQR: 2.5–11) days in low, moderate, and high group, respectively (*p* = 0.11); whereas in liberal strategy, the IC‐LOS was 6 (IQR: 1.5–16), 7 (IQR: 3.5–15), and 5.5 (IQR: 3.5–11.25) days in low, moderate, and high group, respectively (*p* = 0.382) (Table 3).

### 3.3 Length of mechanical ventilation

The LoMV was 1 (IQR: 1–12) day in restrictive and 1 (IQR: 1–15) day in liberal group (*p* = 0.18). Based on Hb increment, low and high increment groups had a median of 1 (IQR: 1–15) and 1 (IQR: 1–16) day of LoMV, respectively (*p* = 0.62). Based on final Hb level, the LoMV was 1 (IQR: 1–7), 1 (IQR: 1–9), and 2 (IQR: 2–6) days in low, moderate, and high group, respectively (*p* = 0.39) (Table 2).

If Hb increment was categorized based on transfusion strategy or initial Hb level, in the restrictive strategy LoMV was 1 (IQR: 1–3) days in low and 1 (IQR: 1–3) days in high increment group (*p* = 0.911); whereas in the liberal strategy, the LoMV was 1 (IQR: 1–5) days in low and 2 (IQR: 1–7) days in high increment group (*p* = 0.358) (Table 3).

If final Hb level was categorized based on transfusion strategy or initial Hb level, in restrictive strategy LoMV was 1 (IQR: 1–3.75), 1 (IQR: 1–3), and 1 (IQR:1–6.5) days in low, moderate, and high group, respectively (*p* = 0.844); whereas in liberal strategy, the LoMV was 1 (IQR: 1–10), 1 (IQR: 1–5.5), and 2 (IQR: 1–6.25) days in low, moderate, and high group, respectively (*p* = 0.567) (Table 3).

### 3.4 Mortality rate

From 65 children who received restrictive transfusion, 40 children were not survive with mortality rates of 61.5%; whereas in liberal transfusion group, 59 from 120 children were not survive with mortality rates of 49.2% (*p* = 0.07). By the Hb increment, the mortality rates in high group were less than in low increment group ([21/50; 42%] vs. [78/135; 58%]; *p* = 0.04), respectively. Based on final Hb level, the highest mortality rate was in low group (16/19; 84.2%), followed by moderate (61/115; 53%) and high group (22/51; 43.1%) (*p* = 0.01) (Table 2).

If Hb increment was categorized based on transfusion strategy or initial Hb level, in the restrictive strategy the mortality rate was 29 of 43 (67.4%) in low and 11 of 22 (50%) in high increment group (*p* = 0.171); whereas in the liberal strategy, mortality rate was 49 of 92 (53.3%) in low and 10 of 28 (35.7%) in high increment group (*p* = 0.104) (Table 3).

If final Hb level was categorized based on transfusion strategy or initial Hb level, in the restrictive strategy mortality rate was 11 of 14 (78.6%), 28 of 46 (60.9%), and 1 of 5 (20%) in low, moderate, and high group, respectively (*p* = 0.078); whereas in liberal strategy, mortality rate was 5 of 5 (100%), 33 of 69 (47.8%), and 21 of 46 (45.7%) in low, moderate, and high group, respectively (*p* = 0.072) (Table 3).

### 3.5 Multivariate analysis

Multivariate analysis in all groups (before categorization based on transfusion strategy or initial Hb level) revealed mortality rate had moderate correlation with Hb increment (odds ratio [OR] = 0.694, 95% confidence interval [CI] 0.549–0.878; *p* = 0.002) and moderate correlation (OR = 0.642, 95% CI 0.519–0.795; *p* = 0.000) with final Hb level (Table 4).

**Table 4:** Multivariate analysis.

All groups without categorization based on transfusion strategy or initial Hb		
Categories	OR (95% CI)	*p* Value
Hb increment	0.694 (0.549–0.878)	0.002*
Final Hb level	0.642 (0.519–0.795)	<0.001*

After categorization based on transfusion strategy or initial Hb		
Restrictive strategy		
Hb increment	0.754 (0.543–1.049)	0.094
Final Hb level	0.634 (0.431–0.932)	0.021*
Liberal strategy		
Hb increment	0.591 (0.422–0.826)	0.002*
Final Hb level	0.624 (0.457–0.853)	0.003*

Abbreviations: CI, confidence interval; Hb, hemoglobin; OR, odds ratio.

*Significant at *p* ≤ 0.05 level.

After categorization based on transfusion strategy or initial Hb level, in restrictive strategy group revealed mortality rate had strong correlation (OR = 0.754, 95% CI 0.543–1.049; *p* = 0.094) with Hb increment and moderate correlation (OR = 0.634, 95% CI 0.431–0.932; *p* = 0.021) with final Hb level; whereas in liberal strategy group revealed mortality rate had moderate correlation (OR = 0.591, 95% CI 0.422–0.826; *p* = 0.002) with Hb increment and moderate correlation (OR = 0.624, 95% CI 0.457–0.853; *p* = 0.003) with final Hb level (Table 4).

## 4 DISCUSSION

From the result we reported, blood transfusion indicated in all of anemia condition in children with critical illness, regardless of the Hb level when anemia found. The effectiveness of blood transfusion to improve clinical outcome and survivality was similar between restrictive and liberal transfusion strategies. However, it is implicated in this study that it is better to give transfusion as soon as it is indicated in critically ill children, because the higher final Hb level is a predictor of better patient's outcome. The response to the transfusion also important to predict the effectiveness of the transfusion since the increment of Hb level significantly correlate with clinical improvement and survivality. Achieving at least 2.5 g/dl of Hb increment and/or 7.0 g/dl of final Hb level, might reduce the mortality on nonhematologic, well‐nourished, and acute anemia of critically ill children.

Clinical outcome of both restrictive transfusion and liberal transfusion does not have significant differences in this study, showed by similar median of IC‐LOS, length of ventilator usage, and mortality rate. These results differ from similar study from Akyildiz et al., which reported shorter length of stay in patients with restrictive transfusion strategy compared to patients with liberal transfusion. The benefit of restrictive transfusion strategy, as mentioned in this study, is more significant increase of arterial oxygen content (CaO_2_) and perfusion index (PI) achieved by restrictive transfusion.^9^ PI used as a parameter to evaluate the efficiency of transfusion to improve the tissue oxygenation. Higher PI shows better peripheral tissue perfusion.

Critical illness causes circulation failure by decrease of tissue oxygen delivery (DO_2_) and followed by decrease of partial pressure of cellular oxygen (PO_2_). DO_2_ depends on two variables, which is arterial oxygen content (CaO_2_) and cardiac output (CO). In condition like hipoxemia or shock, the increase of oxygen demand cannot be accompanied by DO_2_ raise. To maintain the cellular oxygen uptake, the compensatory mechanism is to increase the extraction rate of oxygen (ErO_2_). This compensation had a limit and in the critical point the extraction rate reaches its peak and cannot compensate less DO_2_ to maintain oxygenation. This condition happens in septic shock, when increase of metabolic demands and disruption of oxygen extraction causes inadequate tissue oxygenation.^10^

The main aim of transfusion is to improve tissue oxygenation, which in turn, maintain organ perfusion and function. In the critical patient, improvement of tissue oxygen delivery can be beneficial to accelerate ventilator weaning.^9^ In this study, restrictive transfusion slightly reduces the length of ventilator usage. Liberal transfusion strategy can bring some complication such as fluid overload and nosocomial infection in patient with immunosuppresion which in turn complicate ventilator weaning. In ventilator usage patient, the transfusion is succees if there is an increase of CO gained from preload addition. The weaning process will limitate the tissue oxygen delivery and the compensation is to increase the respiratory muscle work. This process gives an addition of metabolic demands, which must be overcome by CO raise.^11^ There was also a nonsignificant differences of mortality rates between restrictive transfusion group and liberal transfusion group. This result had a similarity with previous study from Hirano et al.^12^ which reported equal rates of mortality in children with restrictive and liberal transfusion.

From the multivariate analysis, there was a significant correlation of Hb increment and final Hb level to mortality rates. After data segregation between restrictive and liberal transfusion, the multivariate analysis showed significant correlation in all, but Hb increment in restrictive transfusion. This result parallel with the previous study from Hogervorst et al.^13^ and Spolverato et al.^14^ who reported the significant decrease of Hb level magnifies the risk to develop complication after surgery. Dropping Hb level of more than 50% can cause transient ischemic attack, cerebrovascular disease, myocardial infarction, and kidney failure. Some factor contribute to the fall of Hb level was the bleeding process and cristalloid infusion throughout surgery.^13^, ^14^ According to WHO, the recommendation of PRC transfusion volume is on 20 ml/kgBW, which can be predicted to increase Hb level for 2.5–3.3 g/dl. This amount of transfusion could minimize complication risk, which of caused by excessive transfusion.^15^

There are some limitations in this study, considering the research design of analytic observational depends on patient's medical records. There is no data for Hb level on prehospital situation, so there is a chance of bias for patient's anemia condition. We also did not include the volume of transfusion in the data, thus the differences of clinical outcome which may be elicited by the amount of transfusion not being analyzed in this study. Future study with more samples or clinical trial design is needed to define the better transfusion for critical illness children in various disease and baseline condition.

## 5 CONCLUSION

From this study, we can conclude the restrictive strategy and the liberal transfusion strategy have a similar effect to IC‐LOS, LoMV, and mortality rate of nonhematologic, well‐nourished, and acute anemia critically‐ill children. The significant increase (≥2.5 g/dl) and moderate‐high final (≥7.0 g/dl) of Hb level after transfusion improve the clinical outcome and reduce the mortality rate, but does not reduce the length of stay and LoMV.

## AUTHOR CONTRIBUTIONS

**Saptadi Yuliarto**: Conceptualization; writing – original draft. **Luluk Nur Azizah**: Investigation; formal analysis. **Kurniawan Taufiq Kadafi**: Investigation; writing – review and editing. **William Prayogo Susanto**: Formal analysis; writing – review and editing.

## CONFLICT OF INTEREST

The authors declare no conflict of interest.

## TRANSPARENCY STATEMENT

The lead author Saptadi Yuliarto affirms that this manuscript is an honest, accurate, and transparent account of the study being reported; that no important aspects of the study have been omitted; and that any discrepancies from the study as planned (and, if relevant, registered) have been explained.

## Data Availability

The authors confirm that the data supporting the findings of this study are available within the article.
